# Filatows or Koplik's Spots

**Published:** 1900-10-06

**Authors:** 


### FILATOW'S OR KOPLIK'S SPOTS.

In the Medical Supplement to the Annual Report of the Metropolitan Asylums Board, there is an interesting paper by Mr. Falkener, assistant medical officer, Western Hospital, on the diagnosis of morbilli by means of the specific spots in the mouth which have been described first by Filatow, and since then more particularly by Koplik.

Many people have doubted the utility and reliability of this diagnostic sign, but Mr. Falkener is able to say that in the 57 cases in which he has looked for them on the days when they ought to be found he has never failed to discover them. There seems to be some discrepancy among different observers as to the earliest moment at which they may be visible, but Mr. Falkener, although on the look-out for them in an infected ward, has not seen them earlier than three days before the rash. The days, however, on which they are certain to be found, if carefully looked for, are the first day of the rash and the day before that. "Whenever the mucosa has been examined for them they have always been in evidence the day previous to the exanthem." Then again in all the cases which were observed on the first day of the rash the spots were present. In some mild cases they disappeared after the first day; but, speaking generally, the spots were nearly always present on the first and second days of the rash. After that, if present at all, they were quite faint.

In regard to their nature, the spots are really fine papillæ, the epithelium on the summits of which has become pulpy and whitened. If they are few in number, the most likely place to find them is opposite to and on a level with the bases of the lower milk molars on either side, where they often form a small cluster, while next to the buccal mucosa the inner surface of the lower lip is the site most commonly affected.

As to their diagnostic utility, Mr. Falkener's observations confirm those of other writers in showing that they are peculiar to measles. He says: "Excluding the presence of morbilli, I have never seen them in any of the 2,000 or more mouths that I have examined during this period," and it must be remembered that these observations were made in a hospital filled with febrile cases of many kinds, just the class of case in which the evidence offered by this sign is important.

Rötheln is perhaps the condition *par excellence* that simulates morbilli, and the one in which the greatest difficulty in differential diagnosis occurs. It is well, then, to remember that Filatow's spots are invariably absent in this disease.

Cases occur in which laryngitis comes on as an early sign or complication of morbilli. It may be of a marked character, with much recession, and such cases are frequently certified and sent in as cases of laryngeal diphtheria. "But if on examination these spots are seen, one can at all events be quite sure of one thing, and that is, that, whatever else the child may have, it most certainly has morbilli," even though no rash be present.

Another class of case may occur in which it may be very important and at the same time very difficult to make a diagnosis; namely, in which morbilli runs its course without the occurrence of any distinctive rash. In such cases the spots may give the necessary clue to the real nature of the disease.

To assure oneself of the nature of these spots, it is necessary to have a good light and to make a careful inspection; to have a correct knowledge of their period of incidence and duration; and an adequate acquaintance with the conditions with which they might be mistaken; but, says Mr. Falkener, "for myself, whenever I have felt definitely sure of the identity of the spots seen with those of Filatow, the further course of events has invariably confirmed the diagnosis; and I may further say that every case of morbilli I have seen since May, 1898, if seen on the appropriate days, has invariably presented these spots."

The result of these observations is to make it quite evident that in Filatow's or Koplik's spots we have a means of diagnosis which will prove of great assistance to the general practitioner, who often finds himself in a position of great difficulty in regard to the early diagnosis of morbilli.

